# Endothelial Cell Loss After Phacoemulsification in a Romanian Cohort: Early Outcomes and Associated Risk Factors

**DOI:** 10.3390/diagnostics16101468

**Published:** 2026-05-12

**Authors:** Aurelian Mihai Ghiță, Daniela Adriana Iliescu, Larisa Adriana Ilie, Ana Cristina Ghiță

**Affiliations:** 1 Physiology Department, Carol Davila University of Medicine and Pharmacy, Bd. Eroii Sanitari No. 8, 050474 Bucharest, Romania; mihai.ghita@umfcd.ro; 2 Ophthalmology Department, Bucharest University Emergency Hospital, Splaiul Independenței No. 169, 050474 Bucharest, Romania; 3 Ocularcare Ophthalmology Clinic, Bd. Ion Mihalache No. 128, 012244 Bucharest, Romania; larisaadriana1907@gmail.com (L.A.I.); eyeana.ghita@gmail.com (A.C.G.)

**Keywords:** phacoemulsification, corneal endothelial cell, cataract surgery, risk factors

## Abstract

**Background/Objectives**: Corneal endothelial damage remains a key concern following phacoemulsification. This study aimed to quantify early postoperative changes in endothelial cell density metrics after cataract surgery in a Romanian population and to identify preoperative and intraoperative predictors of endothelial cell loss at 1 week and 1 month postoperatively. **Methods**: We conducted a retrospective observational study of 137 eyes that underwent standard phacoemulsification with intraocular lens implantation at Ocularcare Ophthalmology Hospital in Bucharest, Romania. Preoperative data included age, sex, and biometric parameters: anterior chamber depth (ACD), axial length (AL), and central corneal thickness (CCT). Corneal endothelium was assessed by specular microscopy preoperatively and at 1 week and 1 month postoperatively, with measurements of endothelial cell density (CD), number of analyzed cells (No), average cell size (ACS), minimum and maximum cell size (MinCS, MaxCS), and cell size variability (SD). Intraoperative parameters included average ultrasound energy (AVE) and actual phacoemulsification time (APT). Associations between demographic, biometric, and surgical variables and postoperative endothelial changes were analyzed using univariable and multivariable regression models. **Results**: In 137 eyes, mean CD decreased from 2401.99 ± 342.57 cells/mm^2^ preoperatively to 2144.38 ± 449.92 at 1 week and 2053.15 ± 471.13 at 1 month. CCT increased from 534.64 ± 38.01 µm to 548.70 ± 41.34 µm at 1 week and remained higher than baseline at 1 month (545.67 ± 42.91 µm). Endothelial remodeling was reflected by significant increases in ACS, MaxCS, and SD, while No and MinCS showed no significant change. In adjusted models, lower postoperative CD was independently associated with shallower ACD and lower baseline CD at both timepoints, whereas higher AVE was associated with lower postoperative CD at 1 week but not at 1 month; sex was not independently associated with postoperative CD. **Conclusions**: In this Romanian cohort, phacoemulsification was associated with significant early endothelial cell loss measurable within the first postoperative month. The magnitude of CD reduction was influenced by both baseline patient ocular characteristics (ACD) and intraoperative phacoemulsification parameters, particularly ultrasound energy (AVE). These findings support the incorporation of preoperative biometric assessment and intraoperative ultrasound minimization strategies to reduce endothelial risk.

## 1. Introduction

Cataract extraction remains the most frequently performed elective operation in ophthalmology worldwide and is highly effective at restoring vision and reducing cataract-associated vision loss. Even with current phacoemulsification platforms and refined surgical techniques, adverse postoperative outcomes may still occur. The corneal endothelium is especially sensitive to intraoperative mechanical and ultrasound-related stress and has negligible proliferative capacity. As a result, endothelial cell loss is compensated predominantly by lateral cell displacement and hypertrophy rather than true regeneration, leading to distortion of the regular hexagonal mosaic, greater morphologic variability, and a net reduction in corneal endothelial cell density (CD) [1,2,3,4]. Clarifying which baseline characteristics and intraoperative parameters are associated with early postoperative CD decline is important for improving perioperative risk assessment and guiding endothelial-protective surgical approaches.

Endothelial cell loss after phacoemulsification is multifactorial. Patient-related determinants such as increasing age, sex, and systemic comorbidities (notably diabetes mellitus) have been associated with greater endothelial vulnerability [1,5,6,7,8]. Ocular conditions that reflect reduced endothelial reserve or impaired endothelial function—such as Fuchs endothelial corneal dystrophy or pseudoexfoliation syndrome—further increase the risk of clinically meaningful postoperative endothelial compromise [9,10,11,12,13,14]. In addition, baseline anatomic and biometric factors may modify intraoperative endothelial exposure. A shallow anterior chamber depth (ACD) can reduce the working distance between surgical maneuvers and the posterior cornea and has been associated with corneal endothelial cell loss in cataract surgery [1,15,16]. On the other hand, central corneal thickness (CCT) is frequently considered an indirect marker of endothelial functional reserve, particularly in eyes with subclinical endothelial dysfunction [17,18,19]. In the present study, we focused specifically on structural endothelial changes rather than on functional endpoints such as visual recovery; however, these early structural alterations remain clinically relevant as they reflect endothelial reserve and the cornea’s ability to tolerate surgical stress during phacoemulsification.

Intraoperative determinants are especially relevant because they are potentially modifiable. Multiple studies have shown that longer ultrasound exposure and higher delivered phacoemulsification energy correlate with greater CD decline, reflecting both direct mechanical and thermal effects and the cumulative burden of intraocular manipulation in more complex cases [2,9,20,21]. Accordingly, surgical metrics that capture ultrasound delivery and exposure time—such as average ultrasound energy (AVE) and actual phacoemulsification time (APT) indices—are increasingly used as objective indicators of intraoperative endothelial stress. Additional surgical complexity factors, including small pupil, prolonged irrigation/aspiration, and intraoperative complications, may further amplify endothelial trauma through increased manipulation and longer surgery duration [14,21]. Although endothelial outcomes after cataract surgery have been widely studied, the magnitude and determinants of early postoperative endothelial change may still vary according to the complexity of the case, timing of surgical presentation, and healthcare setting. In our setting, patients may present relatively late for cataract surgery, potentially with more advanced lens changes at the time of intervention, which supports the value of population-specific evaluation rather than assuming full transferability of findings from other cohorts.

Therefore, the present study aimed to evaluate early structural endothelial changes after uncomplicated phacoemulsification in a Romanian cohort using standardized specular microscopy performed preoperatively and at 1 week and 1 month postoperatively. These early postoperative timepoints were selected to capture the phase of greatest acute endothelial change (within the first month) and the subsequent early stage of postoperative remodeling. CD was defined as the primary outcome, whereas the number of analyzed cells (No), average cell size (ACS), minimum and maximum cell size (MinCS, MaxCS), and cell size variability (SD) were evaluated as secondary endothelial descriptors. We examined the associations of these parameters with demographic variables (age, sex), biometric parameters—ACD, axial length (AL), CCT, and intraoperative phacoemulsification metrics. We hypothesized that lower preoperative endothelial reserve and less favorable ocular anatomy, together with greater intraoperative ultrasound power, would be associated with lower postoperative CD at 1 week and 1 month.

## 2. Materials and Methods

### 2.1. Study Design

This retrospective observational study included 137 patients who underwent uncomplicated cataract phacoemulsification performed by a single surgeon between January 2023 and November 2025. Standard postoperative follow-up was scheduled for 1 day, 1 week, and 1 month. Only cases meeting the predefined inclusion and exclusion criteria at the analyzed time points were included in the final study sample. Predefined inclusion criteria consisted of eyes undergoing uncomplicated cataract phacoemulsification with available baseline and postoperative specular microscopy data, while exclusion criteria comprised combined ocular surgery, any intraoperative or postoperative complication, and missing data at the analyzed time points. Only one eye per patient was included in the analysis. Demographic variables (age, years; sex) were extracted from medical records. All patients underwent a comprehensive preoperative ophthalmic assessment approximately 1 week before surgery, during which ACD (mm), AL (mm), and CCT (µm) were measured using the IOLMaster 700 (Carl Zeiss Meditec, Jena, Germany) in accordance with the manufacturer’s acquisition protocol. Corneal endothelial assessment was performed with an automated non-contact specular microscope (Tomey EM-3000, Tomey Corporation, Nagoya, Japan) at the level of the central cornea at three timepoints: baseline (approximately 1 week preoperatively) and postoperatively at week 1 and month 1. The following endothelial parameters were collected at each time point: CD (cells/mm^2^), defined as the primary outcome measure, together with No (cells), ACS (µm^2^), MinCS (µm^2^), MaxCS (µm^2^), and SD (µm^2^), which were treated as secondary endpoints. All measurements were obtained by a single experienced operator, ensuring consistency in acquisition across all timepoints. The device utilizes automated alignment and image capture with integrated software for endothelial cell analysis, allowing rapid acquisition and automated quantification of endothelial parameters. For each examination, patients were instructed to fixate on the internal target, and central corneal endothelial images were acquired using the auto-alignment function. The system automatically captured multiple images and selected the optimal frame for analysis, a feature consistent with the device’s serial photography capability. To ensure measurement reliability, only images meeting predefined quality criteria were included, namely adequate focus, clear cell borders, and absence of motion or tear film artifacts. In all cases, the automated cell detection provided by the device software was visually inspected and manually verified, and images with obvious segmentation errors, incomplete cell borders, or incorrect cell identification were excluded from analysis. Endothelial analysis was based on a sufficient number of identified cells (typically >100 cells per image), ensuring representative sampling of the corneal endothelial cell layer.

### 2.2. Surgical Technique

Cataract procedures were performed under topical anesthesia using lidocaine gel and oxybuprocaine hydrochloride eye drops, in accordance with standard ophthalmic practice. Surgery was conducted with the Stellaris Elite^®^ Phacoemulsification System (Bausch & Lomb, Bridgewater, NJ, USA). A standardized microincision technique was used, consisting of two paracenteses and a 2.2 mm clear corneal main incision. The operative sequence followed conventional phacoemulsification steps: continuous curvilinear capsulorhexis, hydrodissection and hydrodelineation, nuclear phacoemulsification, irrigation and aspiration of residual cortex, intraocular lens implantation, and aspiration of the ophthalmic viscosurgical device; wound closure was achieved by stromal hydration (hydrosuture). Phacoemulsification settings were adapted to the surgical stage and nuclear density. For softer nuclei (hardness grades 0–2), a stop-and-chop technique was employed; for harder nuclei (grades 3–5), a quick-chop approach was used. Sculpting was performed using continuous ultrasound at 30% power, an 80 mmHg vacuum, and a 95 cm bottle height. During nuclear fragmentation (chop phase), ultrasound power was set to 40% with 2 pulses/s, 50 duty cycle and 500 mmHg vacuum, using an infusion pressure of 90 mmHg. For fragment emulsification, settings were standardized at 50% phaco power, 50% duty cycle, 80 pulses per second (pps), and 90 mmHg infusion pressure to optimize efficiency. Aspiration of cortical remnants and removal of viscoelastic material were completed using 400 mmHg vacuum and 90 mmHg infusion pressure. Total surgical time ranged from 7 to 15 min, reflecting consistent procedural efficiency across varying cataract densities. Intraoperative phacoemulsification metrics recorded for each case included AVE and APT. AVE, as reported by the Stellaris Elite^®^ system (Bausch & Lomb), represents the mean ultrasound power delivered during phacoemulsification and is expressed in device-specific units corresponding to a percentage of the maximum ultrasound power (range between 0 and 100 with APT (seconds)). AVE provides an estimate of total ultrasound exposure, although it is not directly equivalent to cumulative dissipated energy (CDE) used in other platforms.

### 2.3. Statistical Analysis

All statistical analyses were performed using IBM SPSS Statistics (version 26.0; IBM Corp., Armonk, NY, USA). Continuous variables are reported as mean ± standard deviation and categorical variables as counts (percentages). A two-sided *p* < 0.05 was considered statistically significant. Sex-related differences in baseline biometric and endothelial parameters were evaluated using independent-samples *t*-tests (with Levene’s test to assess homogeneity of variances). Associations between continuous variables (e.g., age and ACD) were assessed using Pearson’s correlation coefficient (r). To evaluate changes in CD and other specular microscopy parameters across three repeated measurements (baseline, 1 week, 1 month), general linear models were applied. Mauchly’s test of sphericity was used to assess the sphericity assumption; when needed, Greenhouse–Geisser correction was applied. Post hoc pairwise comparisons between time points were performed using a Bonferroni adjustment for multiple comparisons. To assess whether correlations among the independent variables could have affected the stability of the multivariable regression models, multicollinearity diagnostics were examined for all predictors included in the 1-week and 1-month postoperative CD analyses. This assessment was based on tolerance and variance inflation factor statistics.

## 3. Results

### 3.1. Baseline Characteristics of the Study

The study included 77 females (56.2%) and 60 males (43.8%). Females were, on average, slightly older than males (73.42 ± 8.45 vs. 70.20 ± 10.19 years, respectively). Overall, the mean ACD was 3.03 ± 0.45 mm, and the mean CCT was 534.64 ± 38.01 µm. Baseline specular microscopy showed a mean CD of 2401.99 ± 342.57 cells/mm^2^, mean No of 213.15 ± 50.54 (cells), mean ACS of 427.62 ± 78.51 µm^2^, mean MinCS of 118.67 ± 80.73 µm^2^, mean MaxCS of 1082.42 ± 361.01 µm^2^, and mean AL of 23.29 ± 0.90 mm (Table 1). The cohort had a mean age of 72.01 ± 9.36 years.

Compared with females, males exhibited a significantly deeper ACD and longer AL. CD was higher in males, but the difference did not reach statistical significance (Table 1 and Table 2). CCT did not differ between sexes.

In bivariate analysis, age showed a statistically significant inverse association with ACD (Pearson’s r = −0.184, *p* = 0.031). This indicates that older patients tended to have slightly shallower anterior chambers, although the magnitude of the association was small. Intraoperative phacoemulsification metrics were as follows: AVE (unit as reported by the device) 4.18 ± 3.24 (range 0.00–20.11), and APT 21.17 ± 9.98 s (range 0.00–68.70 s).

### 3.2. Temporal Changes in Endothelial Cell Parameters Evaluated Through Specular Microscopy After Phacoemulsification

Specular microscopy demonstrated significant time-dependent changes in several corneal and endothelial metrics over the early postoperative period (baseline, 1 week, and 1 month). CD decreased progressively from 2401.99 ± 342.57 cells/mm^2^ at baseline to 2144.38 ± 449.92 cells/mm^2^ at 1 week and 2053.15 ± 471.13 cells/mm^2^ at 1 month, with Bonferroni-adjusted pairwise comparisons confirming significant reductions for baseline vs. 1 week (mean difference 257.61 cells/mm^2^), baseline vs. 1 month (mean difference 348.84 cells/mm), and 1 week vs. 1 month (mean difference 91.23 cells/mm^2^). When expressed as percentage change from baseline, endothelial cell density decreased by approximately 10.72% at 1 week and 14.52% at 1 month. In parallel, CCT increased from 534.64 ± 38.01 µm preoperatively to 548.70 ± 41.34 µm at 1 week and remained elevated at 1 month (545.67 ± 42.91 µm), with significant increases from baseline at both postoperative visits; the difference between 1 week and 1 month was not significant. The number of analyzed endothelial cells (No) did not show statistically significant changes across timepoints. However, substantial variability was observed at postoperative assessments, particularly at 1 week and 1 month. This increased dispersion likely reflects transient corneal edema. Accordingly, morphometric parameters derived from automated cell analysis, like No, should be interpreted with caution in the postoperative setting. Consistent with endothelial remodeling, ACS increased significantly from 427.62 ± 78.51 µm^2^ at baseline to 489.85 ± 120.53 µm^2^ at 1 week and 513.20 ± 135.64 µm^2^ at 1 month, with all pairwise comparisons remaining significant after Bonferroni adjustment. Similarly, the SD increased from 174.23 ± 52.23 µm^2^ to 206.58 ± 70.99 µm^2^ at 1 week and to 221.71 ± 75.00 µm^2^ at 1 month, indicating increasing postoperative cell-size variability. MaxCS also increased significantly from 1082.42 ± 361.01 µm^2^ at baseline to 1261.12 ± 483.81 µm^2^ at 1 week and 1347.23 ± 466.89 µm^2^ at 1 month, whereas the increment from 1 week to 1 month was not significant. In contrast, MinCS showed no significant change across time points (Table 3). Collectively, these findings indicate that early postoperative CD reduction is accompanied by transient corneal thickening and endothelial remodeling, primarily reflected by increases in mean cell area and cell size variability. However, morphometric parameters derived from automated cell analysis, particularly in the early postoperative period, should be interpreted with caution given potential measurement variability.

### 3.3. Predictors of Postoperative Endothelial Cell Density at 1 Week

**Figure 1:**
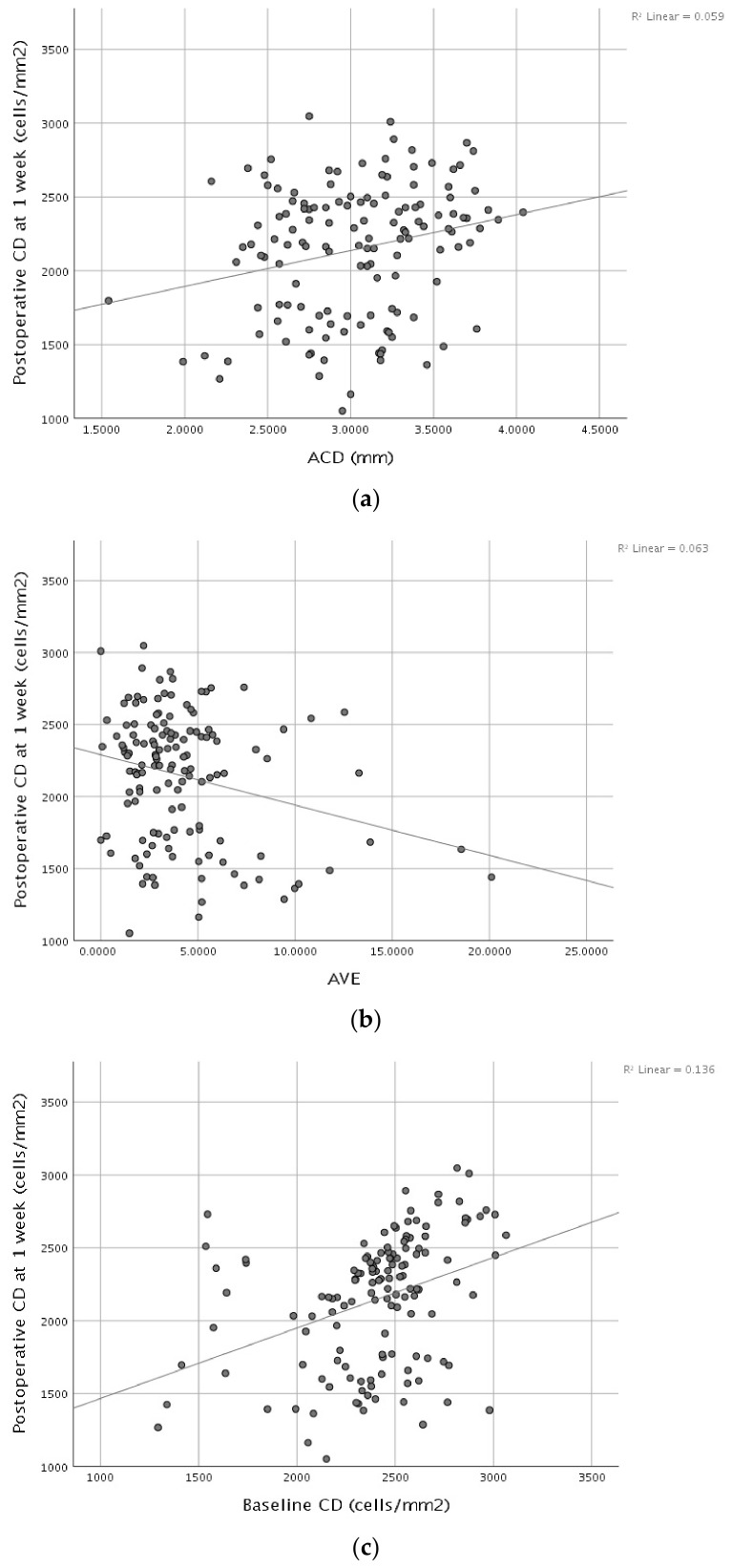
Scatterplots showing the association between postoperative endothelial cell density at 1 week (cells/mm^2^) and (**a**) ACD (mm), (**b**) AVE (device units), and (**c**) baseline CD (cells/mm^2^).

Multiple linear regression was performed to identify independent determinants of postoperative endothelial cell density at 1 week. The overall model was statistically significant and explained 27.2% of the variance in 1-week postoperative CD (R^2^ = 0.272; adjusted R^2^ = 0.245; F = 9.810; *p* < 0.001). After mutual adjustment, baseline CD, ACD, and AVE remained independently associated with postoperative CD at 1 week (Figure 1). Specifically, baseline CD and ACD showed positive associations, indicating that higher preoperative endothelial cell density and deeper anterior chambers were associated with higher postoperative CD at 1 week. In contrast, AVE was negatively associated with postoperative CD at 1 week, consistent with lower CD as delivered ultrasound power increased. Age and APT were not independently associated with postoperative CD at 1 week in this multivariable model. A 1 mm increase in ACD was associated with an estimated 225 cells/mm^2^ higher postoperative CD at 1 week. Each 1-unit increase in AVE corresponded to an estimated 44 cells/mm^2^ lower postoperative CD at 1 week (AVE units as reported by the phacoemulsification platform). Each additional 100 cells/mm^2^ in baseline CD corresponded to an estimated 49 cells/mm^2^ higher postoperative CD at 1 week (Table 4).

### 3.4. Predictors of Postoperative Endothelial Cell Density at 1 Month

**Figure 2:**
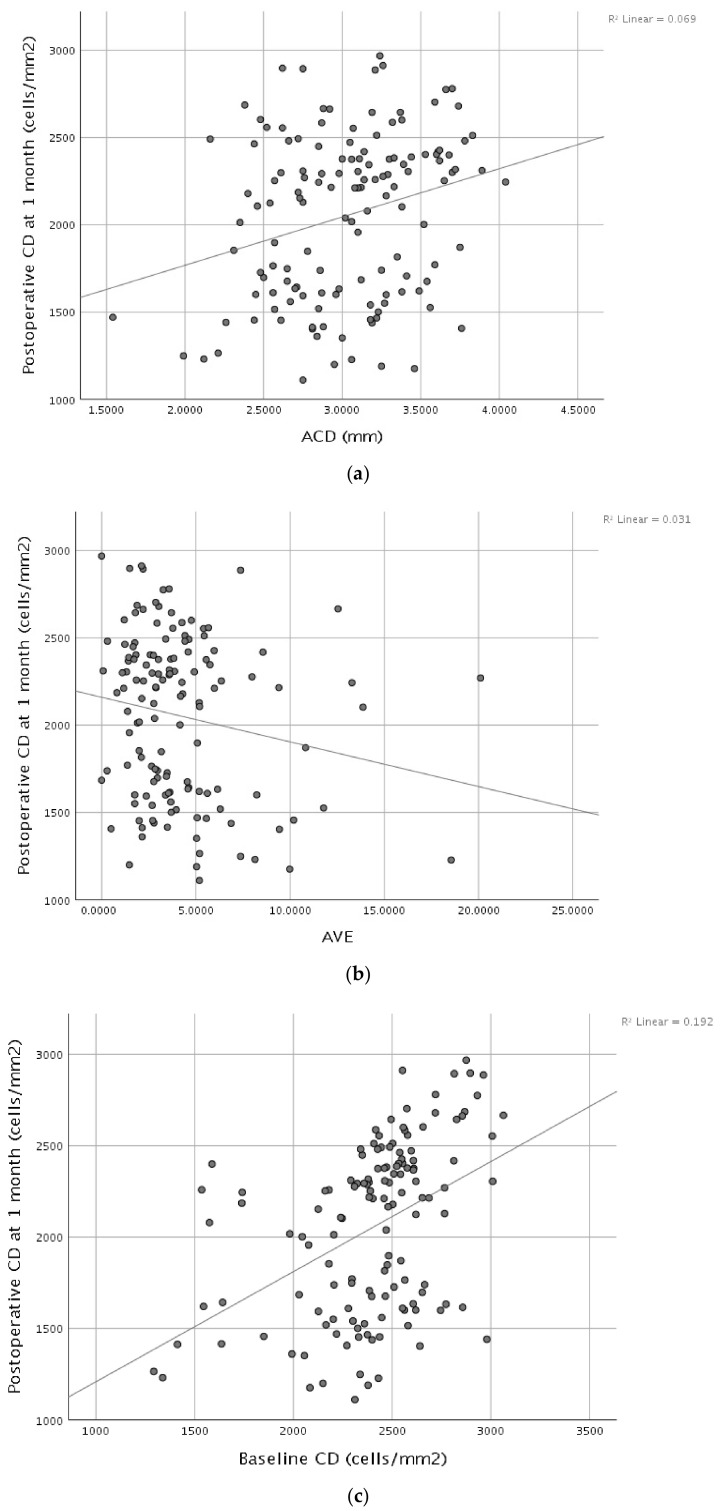
Scatterplots showing the association between postoperative endothelial cell density at 1 month (cells/mm^2^) and (**a**) ACD (mm), (**b**) AVE (device units), and (**c**) baseline CD (cells/mm^2^).

Multiple linear regression was performed to identify independent determinants of postoperative endothelial cell density at 1 month. The overall model was statistically significant and explained 30.7% of the variance in 1-month postoperative CD (R^2^ = 0.307; adjusted R^2^ = 0.280; F = 11.592; *p* < 0.001). After mutual adjustment, baseline CD and ACD remained independently associated with postoperative CD at 1 month (Figure 2). Specifically, both baseline CD and ACD showed positive associations, indicating that higher preoperative CD and deeper anterior chambers were associated with higher postoperative CD at 1 month. In contrast, age, AVE, and APT were not independently associated with postoperative CD at 1 month in this multivariable model. A 1 mm increase in ACD was associated with an estimated 246 cells/mm^2^ higher postoperative CD at 1 month. Each additional 100 cells/mm^2^ in baseline CD corresponded to an estimated 61 cells/mm^2^ higher postoperative CD at 1 month. The magnitude of these effects is clinically relevant, as differences of this scale may influence the risk of postoperative endothelial decompensation, particularly in patients with lower baseline endothelial reserve.

Multicollinearity diagnostics were performed for the multivariable regression models evaluating predictors of postoperative endothelial cell density at 1 week and 1 month. Tolerance and variance inflation factor statistics were examined for all independent variables. Tolerance values ranged from 0.279 to 0.989 and variance inflation factor values from 1.011 to 3.578, indicating no problematic multicollinearity overall. Thus, no major instability of the regression coefficients due to overlap among predictors was identified (Table 5).

## 4. Discussion

In this paper, we provide real-world evidence from an Eastern European practice setting (underrepresented in the literature compared with large Asian or Western academic cohorts) focusing on early (1 week and 1 month) postoperative changes in corneal endothelial morphometry following cataract surgery, thereby capturing the acute phase of endothelial injury after phacoemulsification. This is clinically useful for local counseling, benchmarking, and quality improvement. In our study, the baseline profile of the cohort (mean age ~72 years) is representative of the typical cataract population providing a clinically relevant context for interpreting early postoperative endothelial outcomes. The preoperative CD (~2400 cells/mm^2^) is consistent with commonly reported reference ranges from large specular microscopy series and regulatory reviews, supporting the conclusion that the cohort generally started with an endothelial reserve expected for this age group [22]. Sex-stratified comparisons showed that males had a significantly deeper anterior chamber and longer axial length than females, while central corneal thickness did not differ between sexes. This pattern aligns with population-based biometric data published in other articles, indicating that male eyes have longer axial length and deeper anterior chambers—differences that may be clinically relevant for cataract planning, IOL power selection, and risk stratification in anatomically crowded eyes [23,24]. In addition, the small but significant inverse correlation between age and ACD observed in our dataset is consistent with prior reports showing age-related shallowing of the anterior chamber, plausibly reflecting crystalline lens thickening and anterior segment configuration changes with aging [24,25,26,27].

Regarding intraoperative parameters, the distributions of AVE (mean 4.18 ± 3.24, range 0.00–20.11, device units) and APT (mean 21.17 ± 9.98 s, range 0.00–68.70 s) indicate substantial variability in effective ultrasound time during nucleus management. Although AVE is not directly equivalent to cumulative dissipated energy (CDE), it reflects the mean ultrasound power delivered during surgery and, when considered together with phacoemulsification time (APT), provides a reasonable surrogate of total ultrasound exposure; however, differences in device-specific reporting limit direct cross-platform comparisons. Nonetheless, the broader literature supports the concept that higher ultrasound exposure correlates with increased endothelial stress, reinforcing the clinical value of energy-minimization strategies, particularly in eyes with constrained anterior segment anatomy [28,29,30,31].

In this cohort, phacoemulsification induced a clear early endothelial insult with ongoing short-term change through month 1. The mean CD decrease of ~10.7% at 1 week and ~14.5% at 1 month (relative to baseline) falls within the range commonly reported after uncomplicated phacoemulsification, where endothelial cell loss is frequently cited around 5–20% within the first 1–3 months, depending on case complexity, cataract grade, and energy used [21,32,33,34,35]. Notably, CD continued to decline between week 1 and month 1 in our dataset, supporting the idea that the early postoperative period involves both immediate cell loss and subsequent measurable redistribution rather than a single instantaneous event. Similar early time-course designs (including measurements at ~1 week and later follow-up) have likewise documented significant early reductions in CD after surgery [33,36,37]. The accompanying changes in corneal thickness and morphometrics are clinically coherent with transient endothelial pump dysfunction followed by structural compensation. We observed a modest but statistically significant increase in CCT (~2–3%) at week 1, with partial normalization by month 1, consistent with prior studies describing transient postoperative corneal swelling driven by surgical trauma and endothelial stress, with gradual recovery over subsequent weeks [38,39,40,41,42].

The increase in SD and MaxCS after cataract surgery provides quantitative evidence of polymegathism and compensatory cell spreading. These findings are expected responses given the limited proliferative capacity of corneal endothelial cells. This pattern (lower CD with increased cell size and increased size variability) has been described in the postoperative specular microscopy literature, particularly in studies evaluating short-term endothelial morphology after phacoemulsification [43,44,45].

In contrast, MinCS and No did not change significantly across timepoints in our study. Clinically, these findings are plausible, as early endothelial remodeling typically manifests through the emergence of larger compensatory cells—reflected in changes in average cell size and dispersion—rather than a uniform shift in minimum cell size. The variability observed for the No parameter was substantial at early postoperative timepoints. This increased dispersion likely reflects the influence of transient corneal edema on image quality and automated cell analysis during early postoperative specular microscopy, rather than true biological variation alone. Such variability has been reported in previous studies and represents a known limitation of specular microscopy in early postoperative conditions, particularly when image quality and cell border delineation are suboptimal. Accordingly, these parameters should be interpreted with caution and considered exploratory, with greater emphasis placed on more robust and clinically meaningful indicators such as endothelial cell density, which served as the primary outcome measure [21].

Across both early endpoints, our multivariable models indicate that ACD is an anatomic determinant of endothelial vulnerability after phacoemulsification: shallower chambers were independently associated with lower postoperative CD at 1 week and 1 month, even after adjustment for baseline endothelial status and intraoperative exposure. This finding is consistent with prospective studies that stratified eyes according to anterior chamber depth (ACD). In these studies, eyes with shallower ACD exhibited greater postoperative endothelial cell loss. This observation supports the mechanistic premise that a reduced working distance increases the likelihood that ultrasound turbulence, nuclear fragments, and surgical instrument maneuvers occur closer to the corneal endothelium [1,15,16,21,46].

Our study showed that AVE was negatively associated with postoperative CD at 1 week but not at month 1. The time-dependent behavior of AVE may reflect that ultrasound energy primarily causes an acute intraoperative endothelial insult, which is most readily detected in the early postoperative period. Accordingly, higher AVE was associated with lower postoperative endothelial cell density at 1 week, when the measured values primarily reflect the immediate impact of ultrasound exposure and surgical manipulation. By 1 month, postoperative endothelial cell density reflects a combination of the initial intraoperative injury and subsequent endothelial remodeling, including cell migration and enlargement. At this timepoint, the association with ultrasound energy appears attenuated, likely due to increased biological variability and measurement dispersion inherent to specular microscopy follow-up. In addition, intraoperative ultrasound indices may share variance with other markers of case complexity, such as phacoemulsification time, which may reduce their independent contribution in multivariable models at later timepoints. Consistent with this interpretation, determinants such as baseline endothelial status and anterior segment anatomy (ACD) remained associated with postoperative endothelial cell density at both assessed postoperative timepoints within the first month, whereas the association with ultrasound exposure metrics was evident only at 1 week. [47].

In our study, higher preoperative endothelial cell density was associated with higher postoperative CD at both 1 week and 1 month. Clinically, this finding reflects the role of baseline endothelial reserve, with eyes starting from a higher cell density maintaining better postoperative endothelial status after phacoemulsification. This observation is consistent with previous reports, which also identified baseline endothelial status as an important determinant of postoperative endothelial outcomes. These findings emphasize the importance of preoperative endothelial evaluation in risk stratification and surgical planning [9].

Notably, age and APT were not independently associated with postoperative CD in the fully adjusted models, consistent with evidence that time-based surrogates are weaker predictors when energy delivery and anatomic constraints are considered simultaneously. Even though some studies have found a correlation between gender and CD loss with univariate analysis models, our results do not support them [9,48].

Clinically, our findings suggest that eyes with shallower ACD represent a higher-risk anatomical profile for lower postoperative endothelial cell density. Anterior chamber depth can be easily obtained from routine biometry and may therefore be integrated into preoperative risk assessment and surgical planning. In addition, ultrasound energy appears to influence early postoperative endothelial status, with higher AVE associated with lower postoperative CD at 1 week. Within the first postoperative month, postoperative CD remained associated with baseline endothelial reserve and anterior segment anatomy at both assessed timepoints, whereas the association with ultrasound energy was more evident at the 1-week evaluation.

This study provides a characterization of early postoperative endothelial changes in a Romanian real-world cohort by combining serial specular microscopy morphometrics (baseline, 1 week, 1 month) with multivariable modeling. Nevertheless, this study has several limitations that should be acknowledged. The lack of systematic data on systemic comorbidities (e.g., diabetes mellitus), as well as the absence of endothelial hexagonality measurements in this retrospective dataset, represents a potential source of residual confounding and should be addressed in future prospective studies. The relatively short follow-up period limits conclusions regarding long-term endothelial stabilization and the persistence of observed associations. Also, residual selection bias cannot be fully excluded because of the retrospective single-center design.

## Figures and Tables

**Table 1:** Baseline (preoperative) demographic, biometric, and specular microscopy parameters stratified by sex.

Sex	ACD (mm)	CD (Cells/mm^2^)	CCT (µm)	No (Cells)	ACS (µm^2^)	MinCS (µm^2^)	MaxCS (µm^2^)	AL (mm)
**Female** **(** ***n*** ** = 77)**	2.90 ± 0.44	2356.52 ± 347.30	534.53 ± 41.33	202.00 ± 51.34	437.53 ± 83.07	113.61 ± 57.41	1100.55 ± 351.65	23.10 ± 0.89
**Male** **(** ***n*** ** = 60)**	3.20 ± 0.40	2460.35 ± 330.12	534.77 ± 33.61	227.45 ± 46.07	414.91 ± 70.89	125.17 ± 103.45	1059.17 ± 374.37	23.53 ± 0.86
**Total** **(** ***n*** ** = 137)**	3.03 ± 0.45	2401.99 ± 342.57	534.64 ± 38.01	213.15 ± 50.54	427.62 ± 78.51	118.67 ± 80.73	1082.42 ± 361.01	23.29 ± 0.90

Values are presented as mean ± standard deviation. ACD, anterior chamber depth; CD, corneal endothelial cell density; CCT, central corneal thickness; No, number of analyzed cells; ACS, average cell size; MinCS, minimum cell size; MaxCS, maximum cell size; AL, axial length.

**Table 2:** Mean differences in baseline ocular parameters between sexes with 95% confidence intervals.

Parameter	Unit	Mean Difference (Male − Female)	95% CI of Difference	*p*-Value
ACD	mm	0.2987	0.1541 to 0.4433	<0.001
CD	cells/mm^2^	103.831	−11.927 to 219.588	0.078
AL	mm	0.4320	0.1343 to 0.7298	0.005
CCT	µm	0.2340	−12.757 to 13.225	0.972

Between-sex comparisons were performed using independent-samples *t*-tests; equal variances were assumed based on Levene’s test. Mean differences are reported as **Male − Female** with **95% confidence intervals**. Abbreviations: **ACD**, anterior chamber depth; **CD**, corneal endothelial cell density; **AL**, axial length; **CCT**, baseline central corneal thickness.

**Table 3:** Specular microscopy parameters at baseline, 1 week, and 1 month after phacoemulsification.

Parameter	Unit	Baseline (Mean ± SD)	1 Week (Mean ± SD)	1 Month (Mean ± SD)	*p* (po vs. 1 Week) *	*p* (po vs. 1 Month) *	*p* (1 Week vs. 1 Month) *
CD	cells/mm^2^	2401.99 ± 342.57	2144.38 ± 449.92	2053.15 ± 471.13	<0.001	<0.001	0.002
CCT	µm	534.64 ± 38.01	548.70 ± 41.34	545.67 ± 42.91	<0.001	<0.001	0.572
No	count	213.15 ± 50.54	224.63 ± 318.52	186.42 ± 240.95	1.000	0.611	0.792
ACS	µm^2^	427.62 ± 78.51	489.85 ± 120.53	513.20 ± 135.64	<0.001	<0.001	0.013
MinCS	µm^2^	118.67 ± 80.73	123.55 ± 47.59	128.51 ± 44.24	1.000	0.607	0.748
MaxCS	µm^2^	1082.42 ± 361.01	1261.12 ± 483.81	1347.23 ± 466.89	0.001	<0.001	0.232
SD	µm^2^	174.23 ± 52.23	206.58 ± 70.99	221.71 ± 75.00	<0.001	<0.001	0.015

* Data are presented as mean ± standard deviation. Pairwise comparisons are based on estimated marginal means with Bonferroni adjustment for multiple testing. Abbreviations: CD, corneal endothelial cell density; CCT, central corneal thickness; No, number of analyzed endothelial cells; ACS, average cell size; MinCS, minimum cell size; MaxCS, maximum cell size; SD, standard deviation of cell size; po, preoperatory (baseline).

**Table 4:** Multiple linear regression for predictors of postoperative endothelial cell density at 1 week.

Predictor	B	SE	β	*p* Value
(Constant)	620.04	460.59	—	0.181
Age (VS, years)	−2.97	3.80	−0.06	0.436
ACD (mm)	225.17	76.93	0.22	0.004
Baseline CD (cells/mm^2^)	0.49	0.09	0.37	<0.001
AVE (device units)	−43.79	19.55	−0.31	0.027
APT (s)	−2.44	6.30	0.05	0.699

B, unstandardized regression coefficient; SE, standard error; β, standardized coefficient; ACD, anterior chamber depth; Baseline CD, baseline endothelial cell density; AVE, average ultrasound energy; APT, actual phacoemulsification time (seconds). The dependent variable was postoperative endothelial cell density at week 1 (cells/mm^2^); higher values indicate better endothelial preservation.

**Table 5:** Multiple linear regression for predictors of postoperative endothelial cell density at 1 month.

Predictor	B	SE	β	*p* Value
(Constant)	86.21	470.80	—	0.855
Age (VS, years)	−0.34	3.89	0.00	0.931
ACD (mm)	246.26	78.63	0.23	0.002
Baseline CD (cells/mm^2^)	0.60	0.10	0.44	<0.001
AVE (device units)	−9.04	19.99	0.06	0.652
APT (s)	−8.51	6.44	0.18	0.189

B, unstandardized regression coefficient; SE, standard error; β, standardized coefficient; ACD, anterior chamber depth; Baseline CD, baseline endothelial cell density; AVE, average ultrasound energy; APT, actual phacoemulsification time (seconds). The dependent variable was postoperative endothelial cell density at month 1 (cells/mm^2^); higher values indicate better endothelial preservation.

## Data Availability

The original contributions presented in this study are included in the article. Further inquiries can be directed to the corresponding author.

## Abbreviations

The following abbreviations are used in this manuscript:

ACD	Anterior chamber depth
AL	Axial length
CCT	Central corneal thickness
CD	Corneal endothelial cell density
No	Number of analyzed cells
ACS	Average cell size
MinCS	Minimum cell size
MaxCS	Maximum cell size
SD	Standard deviation of cell size
AVE	Average ultrasound energy
APT	Actual phacoemulsification time
